# Dementia after Ischemic Stroke, from Molecular Biomarkers to Therapeutic Options

**DOI:** 10.3390/ijms25147772

**Published:** 2024-07-16

**Authors:** Vikalpa Dammavalam, Deborah Rupert, Marcos Lanio, Zhaosheng Jin, Neil Nadkarni, Stella E. Tsirka, Sergio D. Bergese

**Affiliations:** 1 Department of Neurology, Stony Brook University Hospital, Stony Brook, NY 11794, USA; vikalpa.dammavalam@stonybrookmedicine.edu (V.D.); marcos.lanio@stonybrookmedicine.edu (M.L.); neil.nadkarni@stonybrookmedicine.edu (N.N.); 2 Renaissance School of Medicine, Stony Brook University, Stony Brook, NY 11794, USA; deborah.rupert@stonybrookmedicine.edu; 3 Department of Anesthesiology, Stony Brook University Hospital, Stony Brook, NY 11794, USA; zhaosheng.jin@stonybrookmedicine.edu; 4 Department of Pharmacological Sciences, Renaissance School of Medicine, Stony Brook University, Stony Brook, NY 11794, USA; styliani-anna.tsirka@stonybrook.edu

**Keywords:** ischemic stroke, cognitive decline, dementia, biomarkers, novel therapy

## Abstract

Ischemic stroke is a leading cause of disability worldwide. While much of post-stroke recovery is focused on physical rehabilitation, post-stroke dementia (PSD) is also a significant contributor to poor functional outcomes. Predictive tools to identify stroke survivors at risk for the development of PSD are limited to brief screening cognitive tests. Emerging biochemical, genetic, and neuroimaging biomarkers are being investigated in an effort to unveil better indicators of PSD. Additionally, acetylcholinesterase inhibitors, NMDA receptor antagonists, dopamine receptor agonists, antidepressants, and cognitive rehabilitation are current therapeutic options for PSD. Focusing on the chronic sequelae of stroke that impair neuroplasticity highlights the need for continued investigative trials to better assess functional outcomes in treatments targeted for PSD.

## 1. Introduction

Stroke is one of the top three leading causes of death and disability worldwide, affecting an estimated 7.6 million per year, with ischemic stroke being the most common subtype [1]. Physical disability from stroke, typically measured with the Modified Rankin Scale (mRS), has been one of the targets for advances in treatment. However, dementia from stroke is a significant contributor to disability as well. Dementia attributable to vascular disease is the second most common cause of dementia after Alzheimer’s disease (AD) [2]. Post-stroke dementia (PSD) is defined as significant, functionally limiting cognitive decline that occurs within 3–6 months following a stroke [3,4]. This is in contrast to post-stroke cognitive impairment, which encompasses any severity of cognitive impairment, regardless of functional impact, which occurs after a stroke [5]. Cognitive impairment after stroke affects up to 40% of stroke survivors, and stroke survivors are more likely to develop cognitive impairment than matched controls [6]. PSD is a subset of vascular dementia (VaD), which additionally encompasses dementia secondary to other vascular issues, including hemorrhage and hypoperfusion, but does not necessarily require a clinically identifiable ischemic event before the development of the cognitive impairment [3].

Stroke increases the risk of dementia two-fold [7]. The prevalence of PSD ranges from 6% to 32% [8]. The PSD incidence is highest within the first 3 months after the initial insult but can develop up to five years later [8]. Symptoms of PSD include aphasia, apraxia, agnosia, impaired executive functioning, behavioral changes, and forgetfulness, but memory deficits are often not the primary manifestation [3,9]. These impairments, coupled with physical disability from stroke, severely impact functional independence and result in increased mortality [2]. Emerging research on biomarkers and current therapeutic options for PSD may improve the quality of life in stroke survivors. We conducted a literature search on PubMed, EMBASE, and Medline for relevant papers published in English between 1990 and 2024 (present). The search terms included the Boolean combinations of “post-stroke dementia”, “post-stroke cognitive dysfunction”, “diagnosis”, “treatment”, and the topic headings. The findings of the literature search were synthesized and narratively reported in the manuscript.

### 1.1. Risk Factors

Patient characteristics, stroke characteristics, and stroke neuro-radiologic features contribute to post-stroke dementia risk factors. Increasing age and pre-existing cognitive impairment are patient characteristics associated with an increased risk of PSD [10,11,12]. Notably, gender does not seem to increase the risk of PSD [10]. Significant vascular comorbidities implicated in stroke, such as hypertension, diabetes mellitus, and atrial fibrillation, were also associated with increased PSD risk [13]. Other recognized risk factors for PSD include baseline cognitive impairment and low functional status [14]. Stroke characteristics such as severity, location, and etiology can influence PSD risk. Increased stroke severity in terms of worse clinical deficit rather than infarct volume was associated with higher rates of PSD 3 months post-stroke [13]. Frontal lobe strokes, bilateral lesions, and left hemispheric strokes confer an increased risk of PSD [15,16]. Atherothrombotic stroke etiology doubled the risk of PSD in those without a prior history of stroke. Cardioembolic etiology increases the risk of PSD in those with a prior history of stroke [7,10]. A history of previous stroke or myocardial infarction was associated with increased PSD risk [10]. Lastly, radiologic findings such as white matter disease and cortical atrophy have been associated with an increased risk of PSD [10,17].

### 1.2. Pathophysiology

The pathophysiology of ischemic stroke is complex [18,19,20,21]. Occlusion of an artery by a clot due to thromboembolism, atherosclerosis, or small vessel disease leads to acute ischemia of the brain tissue supplied by that artery, resulting in hypoxia, failure of oxidative metabolism, and cell death [19,20]. How this acute process leads to the later development of PSD is not well understood, but vascular risk factors, amyloid beta (Aβ), and chronic inflammation are all thought to play a role [14,20,21,22]. In the case of Aβ, A growing body of evidence is showing both an independent and an interactive effect of stroke and Aβ, one of the pathologic hallmarks of AD: Aβ can lead to vascular damage, and tissue hypoxia can lead to increased Aβ deposition, creating a vicious cycle of aggravated ischemia and enhanced Aβ accumulation [20]. The release of intracellular antigens during stroke may also trigger the development of “autoreactive” lymphocytes that promote persistent inflammation of brain tissue, which has been observed in stroke lesions even decades after the event [21]. Thus, the pathophysiology of PSD is seemingly multifactorial, and diverse strategies are needed to address it.

### 1.3. Clinical Testing

Different terminology and criteria have been used in the evaluation and diagnosis of PSD and, more broadly, VaD over the past century. An early study in 1974 coined the term “multi-infarct dementia” for dementia resulting from vascular disease [23]. Given the limitations of this description, multiple additional criteria were published in subsequent years, the most commonly used of which were those of the National Institute of Neurological Disorders and Stroke Association Internationale pour la Recherche et l’Enseignement en Neurosciences (NINDS-ARIEN) international workshop [24], which were intended for research, and the State of California Alzheimer’s Disease Diagnostic and Treatment Centers (ADDTC) criteria [25]. Given the continued heterogeneity in the assessment and diagnosis of patients with ischemic events and dementia, in 2006 NINDS and the Canadian Stroke Network (CSN) convened a panel of experts across the different domains involved in the study, diagnosis, and treatment of these conditions to determine common standards for further research to improve the comparative value of future studies [26]. Shortly thereafter, in 2009, a special symposium of the International Society for Vascular Behavioral and Cognitive Disorders (VASCOG) was held to establish international consensus criteria for diagnosis of what they termed vascular cognitive disorders (VCD) that would be compatible with the forthcoming Diagnostic and Statistical Manual of Mental Disorders Fifth Edition (DSM-5) [27]. Despite these efforts, the adoption of a consensus definition in the field was limited, so a broader international panel was convened for the Vascular Impairment of Cognition Classification Consensus Study (VICCCS), which used a Delphi protocol to determine principles [28] and protocols [4] for diagnosis of what they broadly termed vascular cognitive impairment (VCI).

Putting all the above together, diagnosis of PSD requires:

1. The patient be suffering from sufficiently disruptive cognitive impairment to meet the Diagnostic and Statistical Manual of Mental Disorders (DSM) criteria for Major Neurocognitive Disorder [27],
2. Impairment attributable to a vascular cause [26,29],
3. Development of major cognitive impairment any time after a clinical cerebrovascular event, most commonly assessed at either 3 months or 6 months after incident stroke when possible.

The gold standard for diagnosis relies on a combination of history, clinical exam findings, extensive neuropsychological testing, and supportive imaging findings [29]. Neuropsychologically, VaD patients tend to do relatively better on tests of verbal memory and worse on tests of frontal executive function compared to AD patients [30]. Given the time and specialized training required to conduct this testing, various tools have been developed to screen for and select patients that may necessitate further evaluation [3,31,32,33,34]. Among these, the most widely utilized are the Montreal Cognitive Assessment (MoCA) [33] and the Mini-Mental State Examination (MMSE) [34]. Scores for the MOCA and the MMSE range from 0 to 30, with lower scores indicating increased cognitive impairment [33,34]. Scores below 26 for the MOCA and 24 for the MMSE are generally used as cut-offs below which patients screen positive for cognitive impairment [33,34]. Between these, the MoCA is more sensitive than the MMSE for detecting post-stroke cognitive impairment in both subacute and chronic phases of illness [35,36]. However, aphasia resulting from stroke can be a limiting factor in utilizing these assessments [35,36]. Indeed, the performance characteristics of both these tests, particularly the specificity, are adversely affected by language impairments after stroke [37]. Due to this concern, tools such as the Oxford Cognitive Screen have been developed specifically for stroke patients that are not affected by deficits such as aphasia and neglect [32]. The Oxford Cognitive Screen has not yet been widely adopted but appears to have higher sensitivity with preserved specificity relative to the MMSE [38] and similar sensitivity and specificity to the MoCA [39], although more extensive comparative studies are needed.

## 2. Biomarkers

Predictive tools identifying stroke survivors at risk for the development of PSD would be clinically helpful to mobilize appropriate support resources as well as expand research on treatments and preventive strategies for PSD. Unfortunately, the ability to predict the development of PSD is limited using currently available clinical tools. Pre-existing silent infarcts are a poor predictor of PSD [40]. When used in the acute setting, screening tools such as the MoCA have reasonable sensitivity but relatively poor specificity [41]. In a small study from 2003, researchers developed a logistic regression model using a composite of clinical factors, including age, history of stroke, and severity of impairments at admission, that showed good predictive characteristics [42]; however, it is unclear how widely applicable this model is outside the research population, given differences in demographics and incidence of PSD. Therefore, further research and development of biomarkers are needed to help assess and treat this large and at-risk population.

### 2.1. Biochemical

#### 2.1.1. C-Reactive Protein

C-reactive protein (CRP) is a pentameric acute-phase protein produced in the liver in response to systemic inflammation. CRP induces tau hyperphosphorylation in neuronal cell cultures [43] and, at high concentrations, increases the blood–brain barrier (BBB) permeability in animal models [44]. A hypothesis suggests that neuronal injury and inflammation can induce neuronal production of CRP [45,46], which subsequently enters systemic circulation following BBB disruption in acute ischemic stroke. Choi et al. [47] reported that in patients with ischemic stroke, circulating CRP levels are significantly elevated when compared to patients without underlying neurodegenerative dementia. In patients with ischemic stroke, CRP level during the hospital stay is reported as an independent risk factor for poor functional recovery [48], post-stroke depressive symptoms [49], and mortality [50]. Wang et al. [51] conducted a systematic review and meta-analysis that included nine observational studies and 3800 patients; the authors reported that serum CRP levels were significantly higher in patients with cognitive decline. A prospective study of community-dwelling adults with dementia noted that patients with a history of both stroke and dementia had significantly higher serum CRP levels; however, in patients without a history of stroke, there was no correlation between serum CRP levels and dementia diagnosis. While this is suggestive of a link between serum CRP, stroke, and dementia, the time course of the events is not clear [52].

At the site of inflammation, pentameric CRP irreversibly dissociates into monomeric CRP (mCRP), which has lower water solubility and can accumulate in areas of existing tissue injury and inflammation [53]. The role of mCRP in maintaining a pro-inflammatory microenvironment and perpetuating neuroinflammation has garnered much interest in recent years [54]. Post-mortem histology in patients with ischemic stroke and AD revealed that mCRP is highly expressed in the penumbral regions, as well as in plaques and neurofibrillary tangles [53]. Another study also reported co-localization of mCRP with markers of neuroinflammation such as CD68 and NFκB [55]. However, due to the low water solubility of mCRP, it is mostly tissue bound. mCRP measurement from systemic circulation is of limited value for diagnostic purposes [56].

#### 2.1.2. Interleukin-6

Interkeukin-6 (IL-6) is a pleiotropic cytokine produced in response to tissue injury and inflammation. It subsequently induces the production of other acute-phase reactants, including CRP [57]. Neuronal injury and neuroinflammation can cause the production of IL-6 in the central nervous system [58]. As with CRP, it has been proposed that central production of IL-6 is increased in various neurodegenerative diseases [59], and it may then be released into the systemic circulation during ischemic events and BBB disruption [47]. IL-6 overexpression has been linked to increased TNF-α expression, microglia activation, and cerebellar volume loss [60], corresponding to reduced motor performance. In an observational study of over 10,000 stroke patients, higher serum IL-6 demonstrated a significant correlation with the incidence of recurrent stroke and functional impairment [61].

In AD, higher levels of IL-6 in the brain tissue and the serum were associated with radiological evidence of neuroinflammation and worse performance on neurocognitive testing [62]. A prospective study of over 1000 stroke patients followed for 12 months found that higher serum IL-6 during initial hospitalization was associated with a significantly higher risk of long-term cognitive decline, defined as a reduction of MoCA score by two or more points [63]. Another multicenter study of over 400 ischemic stroke patients reported that higher serum IL-6 during the initial hospitalization was associated with significantly lower MoCA scores at 3 months to 3 years [64]. Overall, increased IL-6 is observed in the setting of post-stroke neuroinflammation, which is associated with worse neurologic outcomes and cognitive impairment.

#### 2.1.3. Matrix Metalloproteinase-9

Matrix metalloproteinase-9 (MMP-9) is expressed by immune, neural, and glial cells, and physiological conditions contribute to neuroplasticity and long-term potentiation [65]. On the other hand, MMP-9 has also been implicated in BBB damage and neuronal injury [66]. Elevated cerebrospinal fluid MMP-9 has been reported in patients with AD and VaD [67]. Higher serum MMP-9 following acute ischemic stroke is also associated with a higher risk of death and significant disability [68]. A more recent study of over 500 patients demonstrated that after adjusting for covariates including age, National Institutes of Health stroke score (NIHSS), and education, higher MMP-9 level within 24 h of initial presentation was associated with significantly higher risk of post-stroke cognitive impairment [69]. Elevated serum MMP-9 may persist for weeks after the initial event and remain related to cognitive recovery [70]. In a study of 317 patients after acute ischemic stroke, those with post-stroke cognitive impairment or PSD have significantly higher serum levels of MMP-9 when compared to those without [71]. Additionally, the authors reported that a single nucleotide polymorphism of the MMP-9 gene, rs3918242, is associated with a significantly higher risk of PSD. MMP-9 mar be a valuable clinical biomarker for risk stratification of PSD, but further studies are needed to validate its predictive value.

#### 2.1.4. Future Prospects on Biochemical Markers

**Figure 1:**
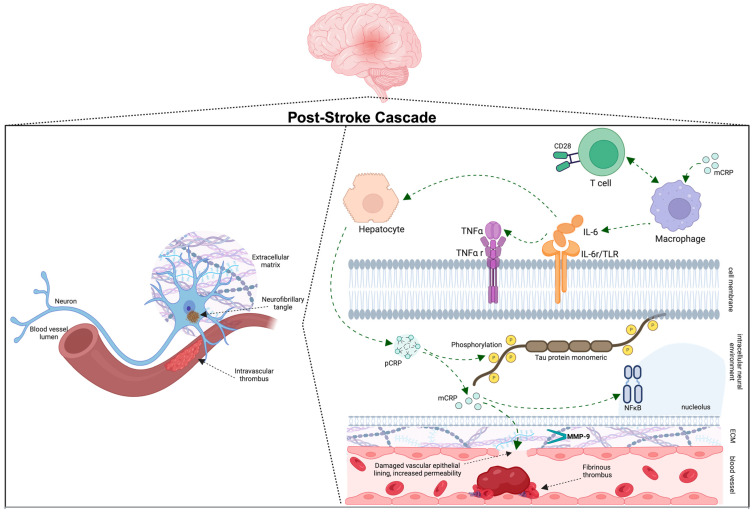
Proinflammatory cascade following ischemic stroke. Pentameric C-reactive protein (pCRP) dissociates into its monomeric (mCRP) form and accumulates in tissues. CRP produced by damaged neurons or hepatocytes induces tau hyperphosphorylation, contributing to the development of neurofibrillary tangles and to blood brain barrier permeability CRP may also contribute to proinflammatory cascades including the TNF-alpha signaling cascade. Cytokines such as IL-6 further induce CRP and TNF-alpha production as well as breakdown of the blood brain barrier. Extracellular metalloproteinase (e.g., MMP-9) activity may also increase following ischemic injury, promoting the degradation of the proteoglycan network that supports the neural synapses.

Figure 1 outlines the interaction of the aforementioned biochemical markers; however, additional putative biochemical biomarkers continue to emerge. Higher levels of serum lipoprotein-associated phospholipase A2 in ischemic stroke patients have been identified as a significant risk factor for VaD as a whole [72]. Increased serum D-amino acid oxidase levels were correlated with white matter changes. Additionally, PSD patients were found to have higher levels of serum D-amino acid oxidase, suggesting a role in PSD diagnosis [73]. Another study found increased urine formaldehyde levels resulted from an overexpression of serum semmethoicarbazide-sensitive amine oxidase in PSD patients and correlated with lower MMSE scores, suggesting its role as a noninvasive predictive test [74]. An ongoing prospective cohort study, The Determinants of Dementia After Stroke (DEDEMAS) trial, uses serum and cerebrospinal fluid analyses, biometric measures, and multimodal imaging to identify PSD biomarkers [75]. Further studies hold promise for potential biomarkers.

The genetic contribution to PSD is incompletely characterized. An early population-based, case-control study comparing the APOE allelic distribution of patients with stroke and concurrent dementia and age and ethnicity-matched controls with stroke but without dementia found that e4 homozygous individuals had a seven-fold increase in dementia risk and heterozygotes had a two-fold increased risk [76]. Of note, this study only looked at prevalence, did not assess the interaction of risk factors, and did not clearly define the stroke or vascular disease burden in controls [76]. Subsequent studies have addressed these points but have not fully borne out these associations [77,78,79]. A smaller prospective study looked at polymorphisms in multiple genes (APOE, ACE, and ACT) that have previously been associated with VaD in patients with incident dementia defined clinically three months post-stroke and found no association between any of the genes and incidence of stroke [77]. A much more significant, population-based cohort study reported that APOEe4 and stroke were independent risk factors for dementia but found no interaction or modification effect between the factors [78], which is in line with a similar previous large study [80]. Lastly, another more recent large population-based cohort study found that only APOEe4 homozygosity was associated with increased risk of both pre- and post-stroke dementia (with post-stroke risk maintained after controlling for baseline cognitive impairment) independent of cerebrovascular burden [79]. Therefore, current evidence indicates that while APOEe4 does increase the risk of dementia, it is not specific to PSD and acts independently of cerebrovascular disease.

### 2.2. Genetic

Other genes associated with stroke and/or VaD risk include angiotensin-converting enzyme (ACE [81]; only stroke), alpha-1 antichymotrypsin (ACT) [82], glutamate-cysteine ligase modifier (GCLM) [83], endothelial nitric oxide synthase (NOS3) [84], and brain-derived neurotrophic factor (BDNF) [85]. As previously noted, in a small prospective study, subsequent research on ACE and ACT did not identify an association with the incidence of PSD [77]. GCLM was initially associated with PSD in a small screening cohort study [83]. Still, a larger subsequent study found a potential protective effect of various GCLM polymorphisms for stroke [86], although this study did not assess cognitive function. In a small prospective study, NOS3 298TT homozygotes had a roughly 3-fold increase in risk of incident dementia [84], although subsequent or larger studies have not confirmed this. Similarly, a small retrospective study showed that BDNF Val66Met carriers developed PSD more quickly than Met/Met homozygotes [85]. However, this association became insignificant after adjusting for other risk factors and has not been further characterized. Taken together, these studies indicate a potential contribution of various genes involved in vascular maintenance and oxidative stress to the development of PSD. Currently, the clinical utility of these genetic findings is limited, and further research is needed to better understand their role and clinical applicability.

### 2.3. Neuroimaging

Neurodegenerative dementia syndromes, particularly AD, have been associated with specific radiologic abnormalities (Table 1) [87,88]. With growing evidence suggesting that the pathophysiology of PSD is not entirely attributed to the initial vascular insult alone, neuroimaging findings such as silent infarct burden, white matter changes, and cortical atrophy have been investigated as potential biomarkers for diagnosis and prognosis in PSD.

Silent infarct burden is regarded as a marker for generalized vascular damage, indicating a high risk of stroke recurrence and overall dementia [89]. Increased number and size of silent infarcts were correlated with a higher frequency of PSD, specifically in the Framingham Study by Ivan et al. [7]. Lacunar infarcts were associated with 4 to 12 times higher likelihood of PSD development regardless of infarct location [90]. Silent infarcts on computed tomography (CT) were correlated with lower mini-mental status examination scores up to 2 points post-stroke [91]. Similarly, a high load of chronic cortical microinfarcts on magnetic resonance imaging (MRI) correlated with slower cognitive recovery, independent of cortical volume and infarct size [92]. Still, a literature review by Henon et al. concluded that global cortical atrophy and white matter changes are better predictors of PSD [93].

Cortical atrophy is associated with an increased risk of PSD [90]. Global and fronto-temporo-insular volume loss on acute MRI obtained 24 to 72 h after initial insult correlated with worse cognitive outcomes on MOCA in the subacute phase up to 1 year later [94]. A later meta-analysis found cortical atrophy and severe white matter changes on acute MRI obtained within 30 days of initial insult to be associated with post-stroke dementia but deemed acute MRI to have uncertain prognostic value in PSD [95]. Hence, medial temporal lobe atrophy, which is a predictive finding of dementia in mild cognitive impairment and is strongly associated with AD, has been a target field of study for identifying additional neuroimaging predictors in PSD [96,97]. A small study comparing the volume of dementia-specific hippocampal subfields on MRI found more atrophy in PSD patients than in nondemented stroke patients but without specific association [98]. A 2009 meta-analysis reported that medial temporal lobe atrophy was strongly associated with pre-stroke dementia resembling subclinical AD but not necessarily associated with PSD [99]. Thus, global cortical atrophy is a more evidence-supported predictor of PSD, while its association with medial temporal lobe atrophy is still under investigation.

White matter changes, or leukoaraiosis, have been associated with higher stroke risk, cognitive impairment, and worse functional outcomes [100,101,102,103]. More notably, white matter changes on MRI were correlated with increased all-cause dementia and increased mortality [104,105]. A prospective observational study found white matter changes to be predictive of early cognitive impairment but were not indicative of functional disability three months poststroke [106]. Although further studies on white matter changes and PSD were limited, a systematic review found white matter changes are associated with PSD as well [107]. A cohort study showed white matter changes were independently associated with pre-stroke dementia and associated with an increased chance of PSD up to 2 years after the initial stroke [108]. Additionally, a case-control study showed that confluent white matter changes with concurrent large stroke were predictive of PSD [109]. Lastly, a recent systematic review concluded that white matter change burden can offer predictive value for PSD and poststroke depression [110].

**Table 1:** Neuroimaging Findings in PSD.

Potential Biomarker	Neuroimaging Description
Silent Infarct Burden	Small, well-defined sub-cortical areas of MRI T2/FLAIR hyperintensity or CT hypodensity consistent with lacunar infarcts without clinical history of stroke [111]
Cortical atrophy	MRI T1/T2 or CT findings showing decreased cortical volume and/or increased ventricular and sulcal spaces out of proportion to age, either globally or most prominent in frontal and temporal regions [112]
Leukoaraiosis	Scattered small areas of sub-cortical MRI T2/FLAIR hyperintensity or CT hypodensity, usually most prominent near the horns of the lateral ventricles, sometimes confluent when more severe [112]

## 3. Current Treatments

### 3.1. Secondary Stroke Prevention

Depending on the purported stroke mechanism, antiplatelet, anticoagulation, and lipid-lowering agents are the mainstay of stroke treatment after acute management with intravenous thrombolytics or endovascular therapy. The pivotal Clopidogrel in High-Risk Patients with Acute Nondisabling Cerebrovascular Events (CHANCE) and Platelet-Oriented Inhibition in New TIA and Minor Ischemic Stroke (POINT) trials demonstrated short-term dual antiplatelet therapy (DAPT) with aspirin and clopidogrel given within 24 h of symptom onset for minor stroke or transient ischemic attack (TIA) is superior to aspirin monotherapy in reducing stroke risk for the first 90 days [113,114]. For cardioembolic minor stroke or TIA due to non-valvular atrial fibrillation, oral anticoagulation is the most effective treatment in reducing stroke recurrence compared to antiplatelet monotherapy or DAPT [115,116]. High-intensity statin treatment soon after stroke or TIA was demonstrated to reduce stroke and cardiovascular events in the hallmark Stroke Prevention by Aggressive Reduction in Cholesterol Levels (SPARCL) study [117]. Unfortunately, the Prevention Regimen for Effectively Avoiding Second Strokes (PRoFESS) trial showed no difference in PSD between aspirin and extended-release dipyridamole versus clopidogrel only [118].

Blood pressure is the most significant modifiable risk factor for stroke [119]. A pivotal prospective study of approximately 1100 hospitalized stroke patients found a U-shaped association between admission systolic blood pressure (SBP) and diastolic blood pressure (DBP) versus mortality at 1 month and 1 year [120]. Specifically, admission SBP of 121–140 was found to be the nadir of the U-shape curve, resulting in the lowest stroke mortality, while higher and lower pressures increased mortality [120]. Furthermore, a population-based observational study showed that poorly controlled blood pressure is an independent risk factor for PSD [121]. The Perindopril Protection Against Recurrent Stroke Study (PROGRESS) trial showed that poststroke blood pressure therapy with perindopril and indapamide reduced the risk of PSD in recurrent stroke [122]. Interestingly, the subsequent PRoFESS trial did not show a difference in PSD incidence in patients with recurrent stroke treated with telmisartan versus placebo [123]. Still, Jiang et al. showed regular use of antihypertensive medications was associated with white matter hyperintensity volume regression, which suggests a decreased risk of PSD [124]. Despite the clear benefits of reducing blood pressure for stroke risk mitigation, the Systolic Blood Pressure Intervention Trial Mind (SPRINT-MIND) randomized clinical trial (RCT) showed intensive blood pressure control of SBP less than 120 in ambulatory adults with hypertension did not significantly reduce the incidence of probable dementia as compared to SBP less than 140, although it did reduce the risk of mild cognitive impairment as well as combined mild cognitive impairment and probable dementia [125]. Recently, a post-hoc analysis from the SPRINT-MIND cohort showed no negative effect of intensive SBP reduction for patients with baseline low DBP [126]. In summary, blood pressure management in stroke and non-stroke patients is likely implicated in mortality rates and development of PSD but more studies are needed to determine the nuances of antihypertensive choice and target blood pressure.

### 3.2. Acetylcholinesterase Inhibitors

Acetylcholinesterase inhibitors (AChEIs) are pharmacological agents that reversibly prevent the breakdown of acetylcholine at the neuromuscular junction and in cholinergic synapses, thereby prolonging synaptic transmission. The mechanisms by which AChEIs are postulated to promote neural plasticity in the setting of ischemic injury center around the replenishment of cholinergic neurotransmission, which may dampen the response to hypoxia and restructuring at cholinergic synapses [127,128,129].

Investigations into AChEIs for the treatment of dementia have largely centered around AD, with the current standard of care dictating a trial of AChEI for AD or Lewy body dementia as a means of targeting executive dysfunction. The role of these agents in VaD and PSD needs to be better established. A meta-analysis of clinical trials including over 4000 VaD patients suggests that AChEIs (donepezil and galantamine) improved cognitive outcomes on the AD Assessment Scale-Cognitive Subscale (ADAS-Cog) with a modest effect size [130]. Another meta-analysis of similar size by Kim et al. suggests a clinically meaningful improvement in cognitive and working memory outcomes as measured by the MMSE and ADAS-Cog after AChEI therapy in VaD and PSD patients [131]. These improvements were most consistently reported as early as 4 weeks of therapy, with continued benefit seen on the ADAS-Cog scale at later follow-up periods up to 24 weeks. Notably, neither team systematically reported the quality of evidence associated with their findings.

The use of AChEIs to specifically address PSD, as distinguishable from multi-infarct or small-vessel forms of VaD, is limited, but safety profiles for early adoption are promising. Among the cholinergic agents, donepezil has been investigated, most specifically in post-stroke patients. In a single-arm, multicenter study, post-stroke patients given donepezil within 24 h of symptom onset showed sound tolerability [132]. These studies report improved cognitive recovery, aphasia, and general functional status, which have been the elusive goal among AChEI trial outcomes [133,134,135]. Small-cohort fMRI data suggest that these improvements may be attributable to the engagement of neuroplasticity mechanisms as part of the “re-wiring” of brain regions involved in these processes [133].

Similarly, several meta-analyses of small randomized clinical trials support the use of rivastigmine in AD and VaD, demonstrating improved behavioral and cognitive symptoms and psychotropic drug-sparing benefits [136]. However, responses to rivastigmine appear to vary between multi-infarct VaD and small vessel VaD etiologies, driving home the possibility that neuroplasticity following acute stroke events varies based on the size and number of total vessels affect and need for subpopulation-focused specific analysis for honing appropriate therapeutic populations [136].

### 3.3. NMDA Receptor Antagonists

Memantine and other N-methyl-d-aspartate (NMDA) receptor antagonists constitute another area of interest because of the role of excess glutamate neurotransmission in excitotoxic neuronal cell death in preclinical investigations. However, clinical data examining NDMA receptor (NMDA-r) antagonists have failed to produce substantial benefit [137]. Failure of both first and second-generation NMDA-r antagonists in post-stroke patients has been attributed to preemptive translation from the bench to clinical trials, quality issues in both study design and therapeutic target specificity as well as the possibility that compensatory mechanisms by neurons accommodate the acute cytotoxic factors [138]. Currently approved for patients with severe AD, the use of these agents in VaD and post-stroke needs to be better established. Clinical investigations into the use of memantine in VaD are limited to short-course interventions; nevertheless, these moderately-sized RCTs of a few thousand patients have resulted in modest improvement in cognitive outcomes [130,139].

Nerinetide, a small peptide that disrupts the NMDA-r cascade through interaction with a post-synaptic protein, has been examined for its potential as a neuroprotective agent in ischemic stroke [140,141,142]. Hill and colleagues led a multi-center RCT in which a little over 1100 patients presenting with acute ischemic stroke were randomized to receive this agent during endovascular therapy or to receive a placebo during that procedure. The primary outcome for this study was improved functional status as measured by the Rankin Scale of neurological disability; secondary outcomes included mortality and NIHSS, all accessed at 90 days of follow-up. No difference was detected. However, a post-hoc interaction between nerinetide and antiplatelet therapy suggests a drug interaction that nullifies the effect of nerinetide. Still, in patients who received tissue plasminogen activator/alteplase, nerinetide was associated with improved outcomes. Therefore, the need to follow up on this particular subgroup of acute ischemic stroke patients and, in particular, to hone in on the cognitive aspects of this potential therapy remains and requires more rigorous investigation.

### 3.4. Dopamine Receptor Agonists

Randomized clinical trial data on the impact of dopamine agonists on post-stroke recovery are mixed. In one meta-analysis, Sami and Faruqui et al. reported the benefit of dopaminergic therapies for recovery of cognitive function but notably combined data from RCTs that enrolled post-ischemic stroke and post-traumatic brain injury patients and represented data from less than 300 cases [143]. However, examining only trials targeting stroke recovery suggests that the effects may be limited to the type of cognitive task. In a comparison of levodopa, methylphenidate, combinations thereof, and placebo in over 1000 patients, Delbari et al. found no changes in MMSE scores. Ramasubbu and Goodyear postulated that the ionotropic and chronotropic properties of methylphenidate contributed to improved intracerebral hemodynamics, leading to better metabolically supported prefrontal and hippocampal networks, as evidenced by functional MRI [144]. The broader potential for stimulants to induce cortical plasticity for enhanced post-stroke recovery has been suggested by several small studies that report improved motor function when combined with physical therapy and initiated early after stroke [145].

A small RCT by Gorgoraptis et al. of 16 patients with hemineglect due to ischemic and hemorrhagic stroke randomized to rotigotine, a high-affinity D1 receptor agonist, demonstrated significant improvement in visual attention, which may reflect improved spatial working memory. Still, no difference was appreciated in tasks that emphasized non-selective, sustained attention [146]. Additionally, treatment had the most benefit in patients with prefrontal damage, highlighting the need to further parse applicability with larger-sized investigations.

### 3.5. Anti-Depressants

There is a high prevalence of depressive symptoms or diagnosis of depression amongst stroke survivors [147,148]. Given the potential for depression to independently contribute to cognitive dysfunction, antidepressant agents appear to improve cognitive dysfunction among patients with post-stroke depression with generally equal efficacy across agent types, including serotonergic and noradrenergic agents [149]. Additionally, independent of treating depressive symptoms, evidence suggests that SSRI therapy (the best studied being fluoxetine and escitalopram) promotes cerebral plasticity, enhancing post-stroke motor function, reaction times, coordination, and other aspects of functional recovery [150,151,152,153]. Antidepressants have also been suggested to support endothelial recovery following stroke, as determined by vascular diameter and flow via ultrasound [154]. The potential for these agents to improve cortical excitability, as measured by functional MRI, leaves open the possibility that such cortical activation would similarly benefit non-motor regions [150,155,156]; however, additional investigations into harnessing these mechanisms to maximize clinical impact remain weak. Additionally, the long-lasting effects of motor rehabilitation in the setting of SSRI therapy suggest that plasticity induced by these agents extends beyond drug cessation [157]. However, the ability of SSRIs to decrease disability is dampened by the lack of change in post-stroke dependence on caregivers [158].

The antidepressants’ role in supporting executive function in stroke patients independent of depression is also unclear as large RCTs are limited. In pursuit of this hypothesis, Narushima and colleagues analyzed data from a small, double-blind, placebo-controlled RCT on 47 stroke patients within 6 months of initial insult [159]. Compared to the initial evaluation of executive function, patients who received antidepressant therapy over 1 year demonstrated improved performance at 21 months, while those who received placebo continued to decline. The type of antidepressant used (nortriptyline vs. fluoxetine) did not affect this outcome, which is a consistent finding across the literature. These findings leave open the possibility of the benefit of SSRI and SNRI therapies to have a lasting impact on cognitive functions, at the very least for maintaining capacities, if not improving upon them, for post-CVA patients. However, untangling the degree to which such findings represent prophylactic prevention of post-stroke depression as compared to supporting post-stroke cognitive recovery is challenging.

In contrast, an RCT by Laska et al. examining 6 months of reversible monoamine oxidase inhibitor (MAO-A-i) therapy for aphasia rehabilitation initiated 3 months post-stroke in 90 patients found no benefit compared to control [160]. Therapy was not combined with speech-language therapy, thereby emphasizing that the potential for therapeutic agents to re-activate neuroplasticity alone is insufficient for rehabilitating complex sensory processing and cognitive processes, as other investigators have suspected [151]. Still, more immediate initiation of therapeutic interventions likely optimizes neuroplasticity. For example, a study showcasing initiation within 4 weeks of stroke demonstrated improvement across outcome measures, including cognitive and functional status [149].

Similarly, MAO-B-is, such as rasagiline and safinamide, are believed to provide neuroprotective properties prior to onset or following injury as evidenced by infarct size [161,162], inflammatory markers [163], and functional recovery [164] in ischemic rodent models. Clinically, these agents have largely been used to support Parkinson’s or AD patients with potentially beneficial cognitive effects for short-duration therapy [165], while metanalysis data suggest little efficacy over longer periods [166,167] in those patient populations. In small clinical studies of less than two dozen patients, the use of MAO-Bi in post-stroke patients has had a limited impact on functional outcomes [168], which suggests that acute timing of potential neuroprotective properties is critical. At this time, additional investigation is warranted, particularly into the development of MAO-Bis with better long-term attenuation of GABAergic activity [169].

### 3.6. Cognitive Rehabilitation

Cognitive impairment predicts the need for inpatient rehabilitation and functional independence [170]. Therapeutic approaches to stroke recovery remain an active area of investigation, with weak or moderate data to support current pharmacological options. Yet, the potential of these agents is likely maximized in conjunction with cognitive rehabilitation. Cognitive rehabilitation is an important aspect of post-stroke care and aims to mitigate deficits and maximize recovery by tailoring multidisciplinary rehabilitation to individual needs. Generally, cognitive rehabilitation emphasizes repetitive exercises and tasks to strengthen neural pathways that support the sensory processing of novel stimuli and executive functions. Cognitive rehabilitation, therefore, includes attention and reaction-time training, logic, reasoning, problem-solving skills, memory testing, and arithmetic. Rehabilitation may also include the identification of compensatory and work-around strategies such as environmental modification and assist devices.

Despite moderately sized RCT data, definitive support for cognitive rehabilitation programs, specifically in post-stroke patients, remains elusive [171,172,173,174]. Tarantino et al. demonstrated that computer-based executive function training improves functional and cognitive performance across domains, including fluency and attention, compared to ordinary rehabilitation alone [174]. There is also growing support for executive function rehabilitation emphasizing working memory and shifting or selective attention-requiring tasks as opposed to the more standard motor, language, and long-term memory practices [175]. These skills have been shown to improve with computer-based interventions, including virtual reality platforms, that readily supplement more standard forms of rehabilitation [174,176,177,178,179]. A small RCT from Kim et al. demonstrated the ability of a virtual reality platform to enhance visual attention, auditory attention, and short-term spatial memory in stroke patients with cognitive impairments [131]. Lastly, cognitive rehabilitation is a long-term process requiring the ability to retrain, compensate, and adapt on a daily basis, indicating the need for continued practice at home after rehabilitation is complete.

### 3.7. Future Prospects in Treatments

A summary of the pharmacologic agents discussed above, though not exhaustive, is provided in Table 2. Nevertheless, to date, no single or combination of therapeutic agents has established unequivocal efficacy for recovery of PSD; the strength of evidence remains weak [180]. Arguably, the strongest data supports a trial of AChEIs to address PSD. However, often these patients presenting with PSD require a multi-modal approach to treatment. For example, the use of stimulants may be implicated for patients presenting with impaired concentration, wherein the lack of sustained attention is thought to contribute to their memory retrieval difficulties. Similarly, anti-depressants are an important adjunct for patients meeting critical for depression in combination with symptoms of dementia, as depression is known to worsen memory challenges. Moreover, many of the agents discussed are believed to promote neuroplasticity, which should be paired with aggressive cognitive rehabilitation work to maximize potential gain.

Additionally, novel investigations of other neuroprotective agents for stroke recovery continue to emerge. Choline-containing phospholipids (CCPLs) are small molecules that act as precursors to the synthesis of phosphatidylcholine, sphingomyelin, and other larger phospholipids and which can transverse the blood-brain barrier. CCPLs are a potential means through which cholinergic transmission can be supported and may provide neuroprotective properties both in bench models and early clinical investigations [181]. In VaD and AD patients, they have been shown to prolong the effects of AChEI therapy [182,183]. However, large, well-controlled studies into using these agents, particularly in the setting of PSD, have yet to take place.

Similarly, preclinical investigation into the ability of phosphodiesterase inhibitors (PDEi) to support cerebral blood-flow following ischemic injury appears promising (REF). As a class, PDEis inhibit the enzyme of the same name, thereby preventing the breakdown of cyclic adenosine monophosphate (c-AMP) or cyclic guanosine monophosphate (c-GMP), depending on the subtype. Preclinical investigation into PDE-5-is has demonstrated angiogenesis properties in addition to inhibition of ischemia-induced apoptosis [184].

There has also been increased discussion on the delivery route for long-lasting neuroprotective agents at the time of revascularization. Hill et al. demonstrated a novel protocol and safety of delivering nerinetide during endovascular therapy [142]. Although no clinical benefit of intra-arterial nertinetide compared to placebo was identified, this study establishes the proof-of-concept potential of targeted intra-arterial delivery of neuroprotective agents if and when a working therapeutic is identified.

**Table 2:** Post-ischemic stroke treatment options for prevention or mitigation of dementia and cognitive impairment.

Pharmacological Category	Example Agents	Mechanisms
Acetylcholinesterase Inhibitors	Donepezil Rivastigime	Enhanced cholinergic neurotransmission [128] Synaptic restructuring [129] Endothelial protection, promotion of revascularization [133] Anti-inflammatory [185]
NMDA Receptor Antagonists	Memantine Nerinetide	Neuroprotection via dampened glutamatergic neurotransmission [137]
Dopamine Agonists	Levodopa Methylphenidate Rotigotine	Improved cerebral hemodynamics [144,145] Enhanced dopaminergic neurotransmission [186] Neuroprotective activity [187]
Antidepressants	Fluoxetine (SSRI) Rasagiline (MAOi)	Prolonged neuroplasticity at serotonergic and/or noradrenergic synapses [156] Enhanced dopaminergic neurotransmission [188] Anti-inflammatory [163]
Choline-containing phospholipids	Citicoline choline alfoscerate	Precursers that feed-forward acetylcholine production and cholinergic neurotransmission [189]

Summary of pharmacological interventions currently under investigation in the prevention and treatment of post-stroke dementia and cognitive impairments. List mechanisms represent suspected means by which plasticity is retained or recovered but not the only effects provided by these agents.

## 4. Conclusions

Post-stroke dementia is a narrow subgroup of VaD distinct from post-stroke cognitive impairment with varied etiology, severity, and symptomology. According to the World Health Organization, the annual global cost of all-cause dementia is approximately US$24,000 per person with dementia [190]. In particular, the cost burden is higher in VaD patients than AD patients by about US$7000 and in non-dementia patients by about US$10,000 [191]. In general, institutional long-term care accounts for the largest portion of the cost burden [192]. The continued search for new diagnostics and treatments is therefore imperative to minimize the cost burden caregivers and PSD patients bear.

Most therapeutic agents for PSD have been studied in heterogeneous enrollment groups and often in concomitant neurological disease states that limit applicability. Still, the search for a novel biomarker, whether biochemical, genetic, or neuroimaging-based, is promising. Reframing stroke as an acute disease with chronic sequelae that impair neuroplasticity can highlight the need for longitudinal and multi-center trials to better assess functional outcomes in treatments targeted for PSD [193].

## Data Availability

No new data was created or analyzed in this study. Data sharing is not applicable to this article.

## Abbreviations

ACE	Angiotensin converting enzyme
AChEi	Acetylcholinesterase inhibitors
ACT	Alpha-1 antichymotrypsin
AD	Alzheimer’s disease
ADAS-Cog	AD Assessment Scale-Cognitive Subscale
ADDTC	Alzheimer’s Disease Diagnostic and Treatment Centers
APOE	Apolipoprotein E
ARIEN	Association Internationale pour la Recherché et l’Enseignement en Neurosciences
Aβ	Amyloid beta
BBB	Blood-brain barrier
BDNF	Brain-derived neurotrophic factor
CAST	Chinese Acute Stroke Trial
CCPL	Choline-containing phospholipids
CHANCE	Clopidogrel in High-Risk Patients with Acute Nondisabling Cerebrovascular Events
CNS	Central nervous system
CRP	C-reactive protein
CSN	Canadian Stroke Network
CT	Computed tomography
DAMPs	Danger associated molecular patterns
DAPT	Dual antiplatelet therapy
DBP	Diastolic blood pressure
DEDEMAS	Determinants of Dementia After Stroke
DSM-5	Diagnostic and Statistical Manual of Mental Disorders Fifth Edition
GCLM	Glutamate-cysteine ligase modifier
IL-6	Interleukin-6
IST	International Stroke Trial
MAO-i	Monoamine oxidase inhibitor
mCRP	Monomeric C-reactive protein
MMP-9	Matrixmetalloproteinase-9
MMSE	Mini-Mental State Examination
MoCA	Montreal Cognitive Assessment
MRI	Magnetic resonance imaging
mRS	Modified Rankin Scale
NIHSS	National Institutes of Health stroke score
NINDS	National Institute of Neurological Disorders and Stroke
NMDA	N-methyl-d-aspartate
NMDA-r	N-methyl-d-aspartate receptor
NOS3	Endothelial nitric oxide synthase
POINT	Platelet-Oriented Inhibition in New TIA and Minor Ischemic Stroke
PRoFESS	Prevention Regimen for Effectively Avoiding Second Strokes
PROGRESS	Perindopril Protection Against Recurrent Stroke Study
PSD	Post-stroke dementia
RCT	Randomized clinical trial
SBP	Systolic blood pressure
SPARCL	Stroke Prevention by Aggressive Reduction in Cholesterol Levels
SPRINT-MIND	Systolic Blood Pressure Intervention Trial Mind
THALES	Acute Stroke or Transient Ischemic Attack Treated With Ticagrelor and ASA for Pre-vention of Stroke and Death
TIA	Transient ischemic attack
VaD	Vascular dementia
VASCOG	International Society for Vascular Behavioral and Cognitive Disorders
VCD	vascular cognitive disorders
VCI	Vascular cognitive impairment
VICCCS	Vascular Impairment of Cognition Classification Consensus Study
